# Physiological stress levels in wild koala sub-populations facing anthropogenic induced environmental trauma and disease

**DOI:** 10.1038/s41598-019-42448-8

**Published:** 2019-04-15

**Authors:** Edward Narayan

**Affiliations:** School of Science and Health, Western Sydney University, Locked Bag 1797, Penrith, New South Wales 2751 Australia

## Abstract

Australian small mammals such as koalas must cope with immense pressure from anthropogenic induced stressors or trauma such as bushfires, vehicle collision impacts and habitat disturbance and land clearance. In addition, they must cope with diseases such as chlamydia. To date, there is no published literature on physiological stress levels in wild koala populations compared with identified environmental stressors. This study investigated physiological stress levels within sub-populations of wild koalas encountering environmental trauma and disease from New South Wales (NSW), Queensland (QLD) and South Australia (SA). Physiological stress was determined using a faecal glucocorticoid (or cortisol) metabolites (FGMs) enzyme-immunoassay (EIA) from 291 fresh faecal samples collected from wild koalas at the point of rescue. A healthy breeding sub-population from a forest reserve in QLD acted as a control group. Clearance of prime Eucalyptus habitat had the largest impact on FGMs, followed by bushfire related factors (e.g. flat demeanour, dehydration and burns injury). Koalas with other sources of physical injury (dog-attacks and vehicle collisions) and disease (chlamydia) also had higher FGMs compared to healthy wild koalas. Healthy wild koalas expressed the lowest median levels of FGMs. Overall, the results highlight that anthropogenic-induced stressors tend to increase physiological stress in wild koalas. Thus, the ultimate stressors such as habitat clearance and bush fire events could increase the incidences of proximate stressors such as dog attacks and vehicle collisions, and increase risks of foliage shortage, diseases and mortality. Therefore, there is need for ecological monitoring, conservation management actions and policy changes to curb the koala population crisis, especially within on-going and future land and road development programs.

## Introduction

Australian wildlife have shown unique survival adaptations to prolonged drought and extreme temperature events^1^, which include decreased metabolism^2–4^, behavioural hiding/movement strategies^5,6^, smaller body size for efficient heat dissipation^7,8^, crepuscular activity and insulating covering^9^. The koala (*Phascolarctos cinereus*), an iconic marsupial, is a unique representation of these. Koalas are marsupial hindgut fermenters able to use low nutrient (50% lignified fibre in *Eucalyptus* leaves^2^), poor quality diet containing essential oils and tannins within an extensive, prolonged, microbial digestive process^2^, highly developed caecum/proximal colon^2^ and basal metabolism 74% of the mean of other marsupials^2^. The koala is listed as nationally Vulnerable^10^ and mortality events vary from vehicle collision impacts to dog attack, particularly young male koalas during natal dispersal, potentially exacerbated by loss of habitat/drought/loss of riparian habitat^5,6,11–14^. Oxalate nephrosis is one of the leading diseases in koalas in the South Australian Mount Lofty Ranges colonies^15^ and chlamydiosis, principally *C*. *pecorum*, is responsible for reproductive consequences, such as urinary and reproductive tract infections^16,17^ in the New South Wales and Queensland colonies. There is evidence of low genetic diversity, before bottlenecks occurred^18^ and appears to reflect a commonality of population instability which in the face of contemporary large scale anthropogenic induced environmental changes may reduce genetic variability of wild koalas^19^ and increases population vulnerability to diseases^18^. Comments made by Spielman, Brook & Frankham^20^ reflected the loss of genetic diversity, inbreeding depression in association with environmental stress/juvenile death increases extinction risk and decreases time to extinction in the conservation status and fragility of the remaining koala colonies around Australia.

Land clearance is one of the significant threats to wild koala populations with a loss of close to 92% of prime Eucalyptus forest and Acacia land cover, which supports some low-density koala populations also reduced to 86% since European settlement in 1800s^21^. In Australia the rates of forest clearing are significant on a global scale^17^. In the state of New South Wales alone, there was a documented reduction in woody vegetation, for the purposes of fire and forestry, of 40,500 ha in 2011–12 and a further 105,900 ha in 2012–2013^22^. For the same years, reduction of woody vegetation for the purposes of cropping, pasture and infrastructure was at a rate of 13,000 ha per year^15,22^. Davies *et al*. (2013) have shown that climate events as seen in south-western Queensland have affected koalas living in the edge of their distribution, where there was an 80% decline in numbers between 1995 and 2009 due to drought^9^. These studies projected that koalas may cope poorly with extreme environment changes such as bushfires and land clearing^9,17^. Most recent ecological analyses conducted on the drivers of koala decline highlighted that disease (chlamydia) and habitat change were two of the major factors influencing koala ecology, however the researchers highlighted that no published study has demonstrated levels of physiological stress generated in wild koalas due to land clearance^23^.

Bushfires are also common in the Australian landscape. The International Union for the Conservation of Nature’s Red List identifies “fire and fire suppression” as a threat to more than 100 threatened species in Australia^24^. Crownfires, which are a forest fire that spreads from tree top to tree top, occur often in south-eastern Australia, where fuel loads are greater, pose direct and indirect threats to the koala. Bushfires directly affect koalas through contact with flames and indirectly when koalas climb smoldering trees and walk across the hot ground. Post-fire losses of food and increased dog attacks on koalas exposed by loss of ground cover represent some of the main indirect effects. Moderate to low intense fires can have significant effects on koala populations through the loss of canopy and food sources. Aside from the direct effects, the resulting habitat loss can also influence their ecological distribution^25^. Researchers have predicted, using bioclimatic modelling^23^, that the current range of the koala will contract eastwards and southwards. This will push koalas into areas where the existing population is already under pressure from threats that imposed by human development, for example, vehicle collisions and dog attacks.

Stress is a hormonal imbalance caused by noxious stimulus in the surrounding or environment of an animal thus making it difficult for the animal to do basic life-history functions. The physiological stress response occurs through acute- and long-term changes in the individual’s environment, which causes activation of neuroendocrine stress system and the release of glucocorticoids (predominantly cortisol in mammals)^26,27^. A physiological stress response could either support behaviour and other basic life-history functions necessary to overcome the stressor (e.g. immune response) or it could be a maladaptive response causing long-term disruption to both physiology and behaviour (especially in the case of chronic stress)^28–30^. The many physiological manifestations of chronic stress affect population performance and hence species longevity^6^. Furthermore, humans rarely are ‘neutral presence’ when studying animals^31^, faecal sampling allows for wildlife physiological stress evaluation with minimal intrusion, using faecal glucocorticoid metabolites (FGM)^32^, does not risk animal injury during capture^33^ and reflects an average and more accurate measure of circulating glucocorticoids^34^ over time as ‘pooling occurs during excretion’^35^. FGMs offer a non-invasive biomarker to quantify physiological stress ensued from environmental stressors in wild koala populations^6^.

The primary aim of this study was to compare FGMs levels across wild koala sub-populations facing known environmental trauma and disease. This study determined which environmental stressor is reflected by the highest FGM concentration in wild koalas. This approach follows the allostasis model^36^ and it proposes that under the influence of moderate environmental stressor(s) there will be significantly higher levels of FGMs recorded in comparison to baseline levels recorded in healthy wild koalas. Studies on chronic stress within a natural wildlife population is limited, however recent work on Asiatic black bears (*Ursus thibetanus*) from rescued bile farms showed that animals living under chronic stress tend to generate significantly higher FGMs levels compared to animals that have been rehabilitated in the bear sanctuary^37^. Thus FGMs levels of koalas in the presence of a moderate environmental stressor (e.g. land clearance) could represent an allostatic overload, which can be verified by comparing the median FGMs levels obtained from healthy koala sub-population.

## Methods

### Approval

All research was done in accordance with the following relevant guidelines and regulations. All current research within New South Wales koalas was formally approved by the Western Sydney University Animal Care and Ethics (ACEC) Committee approval number (Protocol number: A12373). Reference data presented from healthy wild koalas in Queensland were published earlier^38^. Data presented from rescued koalas from South Australia was obtained through a research project formally approved by the Charles Sturt University ACEC Committee (Protocol number: A16044).

### Study sites and Koalas

Wild koala sub-populations in Queensland (QLD), New South Wales (NSW) and South Australia (SA) were studied between the years 2012–2018. Healthy adults (17 males and 12 females) were from a sub-population living in a conserved Eucalyptus forest on the Gold Coast, QLD. Koalas were radio-collared for tracking and re-capture by the Gold Coast City Council and sampled during the summer breeding period of Nov 2011 – February 2012. Some of these koalas carried pouch young during sampling so they also represented a healthy breeding wild koala sub-population.

Koalas studied from NSW were living in a natural habitat that was located within the disturbance zone of land clearance as part of an ongoing state road development study during the sampling period in 2018. Friends of the Koala (FoK) coordinated samples from the land clearance site in NSW and detailed information on the samples and locations are provided in Supplementary Table 1. This site was the largest (156 km length) road infrastructure project in the state of NSW. This study provided an independent assessment of physiological stress levels of the koalas present within the area during the extensive (on-going) road development project. Samples were collected from forest patches present along the cleared areas (covering 16 km transect) with on-going road works and obvious disturbance from heavy vehicle traffic (See Supplementary File 1.0 for data related to sampling transects and dates).

Koalas from SA were rescued wild koalas from the rural-urban areas of Adelaide. Koala Health data were collected in partnership with Adelaide Koala and Wildlife Hospital (AKWH), South Australia. Rescued koalas were sampled opportunistically at the point of rescue in the wild. Healthy male and female koalas were identified as with good body condition (>4.0), no physical signs of disease. Suspected infection cases showed physical signs such as red/swollen/sore eyes/conjunctiva, discharge, red cloaca, wet bottom, swollen genital however tested negative for chlamydia. Other injury included physical injuries sustained from any physical trauma apart from dog-attack or vehicle collision. Burn victims were koalas that were rescued from bush fire impact. Dehydrated patient identified as a koala that was found to be drinking an abnormal quantity of water for an abnormal length of time (e.g. some rescued koalas recorded drinking for over 40 minutes). Flat demeanour was noted when a rescued koala was found in a state of not exhibiting normal behaviours, seemed slow and depressed or was not responding to an external stimulus appropriately. Habitat disturbance was identified as the study site with a patch of few Eucatypus trees with few koalas still present, however the area was heavily disturbed by on-going road development.

In total FGM values of 291 koala samples were included in the data analysis. Information regarding the description of each stressor category is provided in the legend of Table 1. Koala faecal samples collected in the wild underneath harbouring Eucalyptus tree (for QLD and NSW koalas) and at rescue or sighting (for SA and NSW koalas). Fresh (<24 hrs old; see Supplementary Fig. 1 for a natural decay profile of FGMs in koala faeces) faecal pellets were placed into labelled Ziplock^©^ bag and placed into −20 °C storage until analysis within 1 month of sampling. Fresh faeces were immediately frozen to minimise potential effects of sample age on FGM levels^39^. A tag was placed under the sampled tree and scats were cleared to confirm that the sampling was done so that repeated sampling was avoided.

**Table 1:** Faecal cortisol metabolites (FGM) data (ng/g dry weight) for wild koala sub-population represented by known environmental trauma and disease.

	Healthy female	Healthy male	Suspected infection	Other injury	Chlamydia	Dog attack	Vehicle collision	Burn victim	Dehydrated/Kidney issue	Flat on ground	Land clearance
Sample Size	12	17	20	14	120	7	12	5	10	46	28
Minimum	2.47	2.15	6	7	3	14	14	54	22	2	31.95
Maximum	17.99	46.44	36	240	426	47	202	200	144	142	669.90
Median	4.74	9.50	9.50	16	24.50	26	30.50	76	83	88.50	105.90
Fold change in FGMs	7.2	21.6	6	34.28	142	3.35	14.42	3.70	6.54	71	20.96
25 percentile	4.17	4.87	8	10	13	164.96	25.25	59.50	46.25	51.50	55.08
75 percentile	7.21	16.68	15.75	25	50.75	43	45.50	158.5	135	127.50	210.5

Koalas typically have a lengthy delayed lag-time in the metabolism and appearance of FGMs in excreta. In an earlier study, the lag-time of FGMs in koala faeces was confirmed using an ACTH stimulation test, which showed that koalas responded to the ACTH injection with two major peaks, one peak occurred between 24–48 hrs post-injection and a second episodic and delayed peak response was noted to start around 4 days and lasted up to 9 days post ACTH challenge^35^. This delay in response is possibly explainable by the excessively long gut system of the koala^40^. Practically, the first faecal sample used should be representative of FGMs levels of the koala over up to 9 days pre-capture.

A caveat here is that the data was collected from different sites and years so there would have been some influence of natural life-history processes on the koalas during the time samples were obtained. The important note is that each sample was identified to a known environmental stressor. Previous research acknowledged similar caveat in a field study based on assessment of urinary cortisol levels of gray-cheeked mangabeys living in a disturbed forest habitat. The researchers reported that they minimised any capture and handling of the wild animals to avoid risk of chronic stress due to the presence of humans and secondly, they avoided repeated sampling of any one individual in any group, but noted that no tags or collars are on the animals^41^. For each koala faecal sample collected, the environmental stressor was recorded at sampling and used for comparisons of median FGM levels between wild sub-populations.

### Faecal Cortisol Metabolite Enzyme-immunoassays

Laboratory analysis was carried out using methods established earlier^35^. FGM concentrations were determined using a polyclonal anticortisol antiserum (R4866) diluted to 1:15,000 horse radish peroxidase (HRP) conjugated cortisol 1:80,000 and the cortisol standards (1.56–400 pg well^−1^). FGM concentrations were presented as ng/g dry faeces weight basis. Cross reactivity of the R4866 anticortisol antiserum is reported as 100% with cortisol and less than 10% with other steroids tested^42^. The sample assay reagents (supplied by Coralie Munro at the University of California, Davis, USA) have been used for monitoring FCMs in koalas^35^ and other mammals^21,43^. Parallelism for koala faecal extracts on the FGM EIA was reported in earlier study^35^. Assay sensitivity was 2.04 ± 0.3 pgwell^−1^ (n = 20). Intra- and inter- assay coefficients of variation were determined from internal control samples (30% and 70% bound) included in all assays. Intra-assay coefficients of variation were 5% and 3% for low- and high- percentage bound controls, and inter-assay coefficients of variation were 12% and 2%, respectively.

### Statistical Analysis

Median levels of FGMs were compared statistically (representing population level assessment) between each stressor category (environmental trauma and/or disease) using a non-parametric Kruskal-Wallis statistic (Kruskal-Wallis One Way Analysis of Variance on Ranks; See Supplementary File 1 for Raw Data and Statistics Outputs). As the sex of all koalas was not known during sampling from SA and NSW so sex could not be included in the analysis. Sex related differences in FGMs was reported for the healthy wild koalas studied from QLD. Post-hoc comparisons were done using Dunn’s Multiple Comparison Test between each stressor category with both healthy male and female koalas. Statistical analyses were conducted using SYSTAT (version 13.0, Bangalore, India) or IBM SPSS (version 19, NSW, Australia). P < 0.05 was considered significant.

## Results

### Physiological stress in wild koala sub-populations

#### Healthy wild koalas

Healthy wild koalas with no apparent sign of environmental trauma and/or disease recorded median FGMs levels of 4.74 ng/g FGMs level for adult males (n = 17) and 9.50 ng/g FGM levels for adult females (n = 12). Range of FGM levels were 2.15–46.44 ng/g for adult healthy males and 2.47–17.99 ng/g for adult healthy females (p > 0.05)^35^ [Table 1].

#### Wild koalas impacted by environmental trauma and disease

Highest median FGMs levels (Table 1) was recorded for koalas sampled from land clearance area (105.9 ng/g) followed by found flat on the ground (88.50 ng/g), dehydration (83 ng/g), burns from bushfires (76 ng/g), vehicle collision (30.5 ng/g), dog attack (26 ng/g) and injury (16 ng/g). Koalas with chlamydia expressed median FGMs levels at 24.5 ng/g with known infection and 9.50 ng/g with signs of infection. Fold-change in FGMs is also shown for each stressor category in Table 1.

Results showed that overall median FGM levels were significantly different between stressor types [Kruskal-Wallis statistic (H = 248.82, DF = 20, p < 0.0001)]. Post hoc comparison between stressor types shown in the Table 2. Supplementary files (Supplementary File 1, provides all of the raw data used for the statistics including the Kruskal-Wallis statistics output).

**Table 2:** Shows the summary statistics for the Dunn’s Multiple Comparison tests of FGM levels between the various koala sub-populations by stressor categories.

Comparison	Diff of Ranks	Q	P
Land clearance vs wild healthy female	320.345	6.954	<0.001
Land clearance vs wild healthy male	269.590	6.567	<0.001
Land clearance vs suspected infection	256.304	6.557	<0.001
Land clearance vs injury	200.786	4.594	<0.001
Land clearance vs dog-attack	170.250	3.018	0.535
Land clearance vs chlamydia	160.470	5.727	<0.001
Land clearance vs vehicle collision	126.720	2.751	1.000
Land clearance vs flat on ground	41.179	1.287	1.000
Land clearance vs dehydrated/kidney issue	34.429	0.700	1.000
Land clearance vs burn	10.379	0.160	1.000
Burn vs wild healthy female	309.967	4.362	0.003
Burn vs wild healthy male	259.212	3.816	0.028
Burn vs suspected infection	245.925	3.684	0.048
Burn vs injury	190.407	2.737	1.000
Burn vs dog-attack	159.871	2.045	1.000
Burn vs chlamydia	150.092	2.463	1.000
Burn vs vehicle collision	116.342	1.637	1.000
Burn vs flat on ground	30.800	0.490	1.000
Burn vs dehydrated/kidney issue	24.050	0.329	1.000
Dehydrated/kidney issue vs wild healthy female	285.917	5.001	<0.001
Dehydrated/kidney issue vs wild healthy male	235.162	4.420	0.002
Dehydrated/kidney issue vs suspected infection	221.875	4.291	0.004
Dehydrated/kidney issue vs injury	166.357	3.009	0.550
Dehydrated/kidney issue vs dog-attack	135.821	2.064	1.000
Dehydrated/kidney issue vs chlamydia	126.042	2.868	0.867
Dehydrated/kidney issue vs vehicle collision	92.292	1.614	1.000
Dehydrated/kidney issue vs flat on ground	6.750	0.145	1.000
Flat on ground vs wild healthy female	279.167	6.451	<0.001
Flat on ground vs wild healthy male	228.412	6.027	<0.001
Flat on ground vs suspected infection	215.125	6.016	<0.001
Flat on ground vs injury	159.607	3.917	0.019
Flat on ground vs dog-attack	129.071	2.383	1.000
Flat on ground vs chlamydia	119.292	5.152	<0.001
Flat on ground vs vehicle collision	85.542	1.977	1.000
Vehicle collision vs wild healthy female	193.625	3.552	0.080
Vehicle collision vs wild healthy male	142.870	2.838	0.953
Vehicle collision vs suspected infection	129.583	2.658	1.000
Vehicle collision vs injury	74.065	1.410	1.000
Vehicle collision vs dog-attack	43.530	0.686	1.000
Vehicle collision vs chlamydia	33.750	0.835	1.000
Signs of Chlamydia vs wild healthy female	159.875	3.955	0.016
Signs of Chlamydia vs wild healthy male	109.120	3.154	0.338
Signs of Chlamydia vs suspected infection	95.833	2.972	0.621
Signs of Chlamydia vs injury	40.315	1.069	1.000
Signs of Chlamydia vs dog-attack	9.780	0.188	1.000
Dog attack vs wild healthy female	150.095	2.364	1.000
Dog attack vs wild healthy male	99.340	1.657	1.000
Dog attack vs suspected infection	86.054	1.468	1.000
Dog attack vs injury	30.536	0.494	1.000
Injury vs wild healthy female	119.560	2.276	1.000
Injury versus wild healthy male	68.805	1.428	1.000
Injury versus suspected infection	55.518	1.193	1.000
Suspected infection versus wild healthy female	64.042	1.314	1.000
Suspected infection versus wild healthy male	13.287	0.302	1.000
Wild healthy male vs wild healthy female	50.755	1.008	1.000

Results shows the Difference of Ranks, Q statistics and P value (P < 0.05 equals significant comparison).

## Discussion

The use of conservation physiology tools such as non-invasive hormone monitoring allows for the assessment of physiological responses to environmental stressors in wildlife^32^. The application of faecal glucocorticoid metabolite analysis in this study provided an opportunity to assess stress hormone levels in wild koala sub-populations facing environmental trauma and disease. Land clearance and bushfire showed the highest median FGM levels followed by vehicle collision, dog-attack and chlamydia. Wild healthy male and female koalas showed the lowest median levels of FGMs. It is evident that ultimate stressors such as land clearing and bushfires are inducing greater risks to wild koala populations through chronic stress.

In another study, researchers^44^ showed habitat disturbance through logging and fragmentation increased chronic stress (indexed using neutrophil to lymphocyte ratios) and diminished immune function in foliage-roosting paleotropical bat species in Malaysian Borneo. Habitat decimation creates complex ecological problems for wildlife species through reduction in foraging space. Therefore, wildlife tend to move around more frequently in search of food and shelter. This increases risks of predation such as dog-attacks and potential injuries due to vehicle collision (see reviews on how anthropogenic perturbation of the environment can result in chronic stress in wildlife^39,45^). Chronic stress is a state whereby the organisms are no longer capable of adaptation to their environment as normal body functions have been compromised^36,46–48^. Chronic physiological stress can also result in impaired immune system function leading to increased susceptibility, shedding of pathogens and diminished survival rates^49^.

Habitat clearance and climate change can be considered as ultimate stressors that may superimpose numerous underlying or proximate ‘immediate’ stressors. Therefore, in the presence of ultimate stressors it is likely that wildlife populations will have increased risk of immediate stressors especially those associated with nutritional stress^50^, altered social contact rates and changing patterns of parasite infection^38^. Habitat fragmentation may also increase chronic stress through the creation of more habitat edges^44^. In earlier studies, Brearley^22,51^ measured chronic stress (using cortisol metabolites in hair) in Squirrel gliders (*Petaurus norfolcensis*) living in edge habitats and found that cortisol levels were higher compared to their congeners living in interior habitats. In a study based on the health of diademed sifaka (*Propithecus diadema*), reseachers^52^ showed the impact of habitat disturbance on the population viability of the primate population living in a disturbed habitat. The study showed changes in key blood biochemistry parameters such as extremely low blood urea nitrogen (perhaps reflecting protein limitation) and selenium levels in lemurs from disturbed habitat. The researchers also suggested that further assessments of the fitness consequences related to physiological imbalances are required to obtain better picture of the impacts of habitat disturbance on population viability. More conservation resources need to be directed for the protection of wildlife living within disturbed habitats. In another study, researchers^53^ demonstrated that populations of the agile Antechinus (*Antechinus agilis*) living within disturbed forest fragments of Southeast Australia were facing chronic stress. The researchers determined haematological indicators of stress and condition in *A*. *agilis* populations in 30 forest fragments and 30 undisturbed, continuous forest sites (pseudofragments) in south-eastern Australia over 2 years. Results showed chronic stress related changes in haematological parameters such as elevated neutrophil/lymphocyte ratio as well as abnormal changes in total erythrocyte count and haematocrit and mean erythrocyte haemoglobin content associated with stress mediated regenerative anaemia.

The presented flow on effects of environmental ‘state’ are suggestive that koalas living in areas of land clearance are likely to be experiencing increased risk of chronic stress. Environments where vegetation has been recently removed presents the animal occupants with multiple abiotic stressors^46,54^ (e.g.: suboptimal temperatures) and biotic stressors (e.g., predation and nutritional stress). The clearing of land to due to agriculture, urbanisation as well as fragmentation barriers such as roads and towns create gateway to reduced gene flow in threatened species^55^. Habitat fragmentation is increasing susceptibility to a large range of deleterious effects of loss of genetic diversity which is directly linked to population isolation, environmental changes, climatic change and the presence of disease^55^. In a study, researchers^56^ showed that habitat fragmentation is negatively affecting long-term reproductive viability in some koala populations.

Land division (fragmentation) is parallel to the creation of patches. The loss of land creates a large number of smaller habitat patches in a given area therefore a higher density of species in those smaller zones^57^. A study^58^ over a 16-year period (1894–1910) found average increases in koala patches in Queensland rising from 74 to 490 patches and found a decrease in patch sizes from 966 ha to 63 ha. The probability of local extinctions is found to be directly correlated to the functioning of patch size and inter-patch movements^59^. Lowered quality dispersal habitat is of particular concern to species with immediate dispersal abilities such as the koala^13^. This is due to increased competition, a decrease in quality habitat and therefore a reduction in species distribution and occurrence^13^.

Koalas are also specialist folivores, having limits in their choice of resources^55^. Due to being a specialist diet species, they are especially vulnerable to land clearing^55,59^. There has been suggestion that koalas also have foliage preference within eucalypt species^60^. Researchers^61^ noted preference to larger trees that would likely provide added shelter during extreme weather conditions. A study^60^ also looked into eucalypt preference in which Swamp Gum Eucalyptus trees had strong preference but there was also large variation of this between seasons. Researchers^60^ further discussed this with the preference of one type of eucalypt when compared to eight other species. Studies^60^ and^61^ both showed an increasing concern as there is a decrease in eucalypts due to habitat fragmentation and there is also high preference towards food. It is evident that there is then a physical reduction of food resources, which permits an increase in intra-species food competition^62^. The combined effects of food competition and loss of resource correlates to physiological stress and lowers immunity and resistance to diseases^62,63^.

In a recent study published in Science^64^, it was recorded that 80% of the Earth’s surface is roadless, which is divided into 600,000 patches most of which are <1 km square. Suggestions to habitat loss related stress and disease have been proposed in koala populations, particularly in the incidence of the chlamydiosis^17,65^. The devastating effects of chlamydia to koala populations is enormous, often causing urinary tract diseases, reproductive tract diseases, pneumonia and even blindness as a result of conjunctivitis^17^. Where fragmentation creates patches of individuals free of the disease, this fragmentation is likely to reduce disease spread^66^. However, within-patch transmission if there is a pathogen host present will often mass spread due to higher densities of individuals in small areas and the increased susceptibility to disease due to habitat loss^66^.

A study^67^ highlighted the trends in local extinctions in the Australian context in 1994 with some lack of information for more recent periods of time. Almost 50% of extinct mammals worldwide are from the Australian continent^67^. Researchers^68^ evaluated habitat loss and extinction in biodiversity hotspots and concluded that habitat loss to be the leading or in some areas has already led to an extinction crisis. There has been demonstration of local extinction in Australian bird populations as a result of habitat loss. Such extinctions were seen in the loss of Hooded robins in Victoria, close to Armidale, NSW^69^. Similar patterns were seen in local extinctions of the brown treecreeper as well as the crested bellbird in North Eastern Victoria^69^.

Earlier study by^70^, concluded three key findings in the fate of koala species, those being; the current levels of mortality and fertility are no longer able to support the population, that significant improvements in mortality and fertility alone would still unlikely prevent species decline towards extinction and that immigration was one of the key factors in maintaining populations. The current changes in public perception are also at a rate slower than the actual population changes^70^.

### Influence of key factors on FGMs and considerations for future research

An important consideration (caveat) required for explaining the observed variation in FGM levels of wild koalas between the study sites is the potential effects of ‘state versus event’ related stressors and sampling design limitation to match FGMs to acute stressors. For example, a vehicle collision impact or dog attack could be an ‘event’ related acute stressor while ‘state’ related stressor could be an example of land clearance. The methods section described each of the stressor category however, given that, only one faecal sample was obtained for each koala, the results should be interpreted with caution. For example, in the instance of a dog-attack case, FGMs levels within single faecal sample may not be 100% representative of the acute stress (due to the delayed lag-time of the FGMs in koala faeces), especially if the dog-attack occurred within only a few hours prior to rescue. It was assumed in the current study that the levels of FGMs quantified within a sample (taken at the point when a wild koala was rescued and case diagnosed) would provide an accumulative level of FGMs upto several days prior to the rescue. It could be assumed that the dog-attack victim may have been attacked and injured several days prior to being rescued hence in this case the FGM levels from sample taken at the point of rescue would be more representative of the physiological stress generated due to this ‘event’. However, in the absence of exact time-series matched faecal sampling for each stressor category it is important to interpret and use the results with caution.

Furthermore, koalas have unique life-history characteristics, in both males and females. For example, female koalas nurse their joeys for 1 year and they invest heavily on maternal care to increase joey growth and survivorship^71^. Reproduction and maternal effort are proximate stressors for female koalas due to the prolonged time, energy and resources needed to produce and rear the joey. Koala mating system is also very complex and researchers have suggested that male koala characteristics such as age, bellows and scent advertisements are key traits that female koalas use for mate selection^71,72^. Interactions among stressors do exist in the natural world whereby multiple stressors act (either synergistically or antagonistically) to bring a net impact on biodiversity and ecosystem function^73^. Life-history related factors such as sex, age, reproductive status, social dynamics can contribute to the stress endocrine response, which enable wildlife to survive and carry-out essential life-history functions such as foraging, breeding and predator evasion^74^. If extreme natural or anthropogenic induced stressors are introduced to natural systems then it creates additional burden for the wildlife, which could create subtle imbalances in the physiological stress coping and adaptive capacity of wildlife and potentially increase disease risks and mortality^75^.

Therefore, it is important that future research should focus on not just single anthropogenic induced or extreme environmental stressor, however there needs to be an integrative approach to determine the potential interactions (synergism and/or antagonism)^73^ among the natural life-history related stressors and extreme environmental stressors. Through the integration of modern-age techniques such as GPS monitoring of koalas^76^ in combination with non-invasive hormone monitoring^6^ and veterinary clinical health checks^77^, new knowledge can be obtained on the impacts of proximate and ultimate stressors on wild koalas and enable conservation managers to target management efforts within the most vulnerable and chronically stressed populations.

In conclusion, this is the first data set on physiological stress in wild koalas undergoing direct threat from environmental trauma and disease. Conservation decision makers and managers are recommended to acknowledge the physiological status of wildlife species and applications of non-invasive hormone monitoring into species recovery programs should be relatively straightforward because the methods have been robustly validated and tested in various studies. Furthermore, urgent management actions by local government and state is required to attempt to seize the dramatic decline of the koala.

## Supplementary information

Dataset 1

## Data Availability

The Adelaide Koala and Wildlife Hospital have been duly acknowledged for providing clinical data that have been adequately presented in graphs.

## References

[1] Pittock B, Abbs D, Suppiah R, Jones R (2006). Climatic background to past and future floods in Australia. Adv. Ecol. Res..

[2] Cork SJ, Hume I, Dawson T (1983). Digestion and metabolism of a natural foliar diet (Eucalyptus punctata) by an arboreal marsupial, the koala (Phascolarctos cinereus). Journal of Comparative Physiology.

[3] Degabriele R, Dawson T (1979). Metabolism and heat balance in an arboreal marsupial, the koala (Phascolarctos cinereus). Journal of Comparative Physiology B: Biochemical, Systemic, and Environmental Physiology.

[4] Nagy KA, Martin R (1985). Field metabolic rate, water flux, food consumption and time budget of koalas, Phascolarctos cinereus (Marsupialia: Phascolarctidae) in Victoria. Aust. J. Zool..

[5] Black KH, Price GJ, Archer M, Hand SJ (2014). Bearing up well? Understanding the past, present and future of Australia’s koalas. Gondwana Research.

[6] Davies, N., Gramotnev, G., McAlpine, C., Seabrook, L. & Baxter, G. Physiological Stress in Koala Populations near the Arid Edge of Their (2013). 10.1371/journal.pone.0079136 PMC3827162 24265749

[7] Welbergen JA, Klose SM, Markus N, Eby P (2008). Climate change and the effects of temperature extremes on Australian flying-foxes. Proceedings of the Royal Society B: Biological Sciences.

[8] McKechnie, A. E. & Wolf, B. O. Climate change increases the likelihood of catastrophic avian mortality events during extreme heat waves. *Biol*. *Lett*, rsbl20090702 (2009). 10.1098/rsbl.2009.0702 PMC2865035 19793742

[9] Dawson TJ, Webster KN, Maloney SK (2014). The fur of mammals in exposed environments; do crypsis and thermal needs necessarily conflict? The polar bear and marsupial koala compared. Journal of Comparative Physiology B.

[10] McAlpine C (2015). Conserving koalas: A review of the contrasting regional trends, outlooks and policy challenges. Biol. Conserv..

[11] Ellis W, Melzer A, Clifton I, Carrick F (2010). Climate change and the koala Phascolarctos cinereus: water and energy. Aust. Zool..

[12] Gordon G, Hrdina F, Patterson R (2006). Decline in the distribution of the koala Phascolarctos cinereus in Queensland. Aust. Zool..

[13] McAlpine CA (2006). The importance of forest area and configuration relative to local habitat factors for conserving forest mammals: a case study of koalas in Queensland, Australia. Biol. Conserv..

[14] Phillips S, Callaghan J (2000). Tree species preferences of koalas (<emph type = “2”> Phascolarctos cinereus </emph>) in the Campbelltown area south-west of Sydney, New South Wales. Wildl. Res..

[15] Speight KN (2014). Plasma biochemistry and urinalysis variables of koalas (Phascolarctos cinereus) with and without oxalate nephrosis. Vet Clin Pathol.

[16] Morris KM (2015). Identification, characterisation and expression analysis of natural killer receptor genes in Chlamydia pecorum infected koalas (Phascolarctos cinereus). BMC Genomics.

[17] Polkinghorne A, Hanger J, Timms P (2013). Recent advances in understanding the biology, epidemiology and control of chlamydial infections in koalas. Vet. Microbiol..

[18] Tsangaras K (2012). Historically low mitochondrial DNA diversity in koalas (Phascolarctos cinereus). BMC Genet.

[19] Houlden B, England P, Taylor A, Greville W, Sherwin W (1996). Low genetic variability of the koala Phascolarctos cinereus in south‐eastern Australia following a severe population bottleneck. Mol. Ecol..

[20] Spielman D, Brook BW, Frankham R (2004). Most species are not driven to extinction before genetic factors impact them. Proceedings of the National Academy of Sciences of the United States of America.

[21] Narayan E, Hero JM, Evans N, Nicolson V, Mucci A (2012). Non-invasive evaluation of physiological stress hormone responses in a captive population of the greater bilby (*Macrotis lagotis*). *Endangered Species*. Research.

[22] Brearley G, McAlpine C, Bell S, Bradley A (2012). Influence of urban edges on stress in an arboreal mammal: a case study of squirrel gliders in southeast Queensland, Australia. Landscape Ecol..

[23] Adams-Hosking C, Grantham HS, Rhodes JR, McAlpine C, Moss PT (2011). Modelling climate-change-induced shifts in the distribution of the koala. Wildl. Res..

[24] Doherty TS, Glen AS, Nimmo DG, Ritchie EG, Dickman CR (2016). Invasive predators and global biodiversity loss. Proceedings of the National Academy of Sciences.

[25] Melzer A, Carrick F, Menkhorst P, Lunney D, John BS (2000). Overview, critical assessment, and conservation implications of koala distribution and abundance. Conserv. Biol..

[26] Wasser SK (2000). A generalized fecal glucocorticoid assay for use in a diverse array of nondomestic mammalian and avian species. Gen. Comp. Endocrinol..

[27] Muller MN, Wrangham RW (2004). Dominance, aggression and testosterone in wild chimpanzees: a test of the ‘challenge hypothesis’. Anim. Behav..

[28] Sapolsky RM (1986). Glucocorticoid toxicity in the hippocampus: reversal by supplementation with brain fuels. J Neurosci.

[29] Rich EL, Romero LM (2005). Exposure to chronic stress downregulates corticosterone responses to acute stressors. American Journal of Physiology - Regulatory, Integrative and Comparative Physiology.

[30] Whirledge S, Cidlowski JA (2010). Glucocorticoids, stress, and fertility. Minerva endocrinologica.

[31] Jack KM (2008). The effects of observer presence on the behavior of Cebus capucinus in Costa Rica. American Journal of Primatology: Official Journal of the American Society of Primatologists.

[32] Millspaugh JJ, Washburn BE (2004). Use of fecal glucocorticoid metabolite measures in conservation biology research: considerations for application and interpretation. Gen. Comp. Endocrinol..

[33] Mostl E, Palme R (2002). Hormones as indicators of stress. Domest. Anim. Endocrinol..

[34] Harper JM, Austad SN (2000). Fecal glucocorticoids: a noninvasive method of measuring adrenal activity in wild and captive rodents. Physiol. Biochem. Zool..

[35] Narayan E, Webster K, Nicolson V, Mucci A, Hero J-M (2013). Non-invasive evaluation of physiological stress in an iconic Australian marsupial: the Koala (*Phascolarctos cinereus*). Gen. Comp. Endocrinol..

[36] McEwen BS, Wingfield JC (2003). The concept of allostasis in biology and biomedicine. Horm. Behav..

[37] Narayan E, Willis A, Thompson R, Hunter-Ishikawa M, Bendixsen T (2018). Evaluating physiological stress in Asiatic black bears (*Ursus thibetanus*) rescued from bile farms in Vietnam. Anim. Welfare.

[38] Gillespie T, Chapman R, Grenier CA (2005). E. C. Effects of logging on gastrointestinal parasite infections and infection risk in African primates. J. Appl. Ecol..

[39] Romero LM (2004). Physiological stress in ecology: lessons from biomedical research. Trends Ecol. Evol..

[40] Cork SJ, Warner ACI (1983). The passage of digestion markers through the gut of a folivorous marsupial, the Koala Phascolarctos cinereus. Journal of Comparative Physiology.

[41] Jaimez NA, Bribiescas RG, Aronsen GP, Anestis SA, Watts DP (2012). Urinary cortisol levels of gray-cheeked mangabeys are higher in disturbed compared to undisturbed forest areas in Kibale National Park, Uganda. Anim. Conserv..

[42] Munro C, Stabenfeldt G (1985). Development of a cortisol enzyme immunoassay in plasma. Clin. Chem..

[43] Muehlenbein MP (2012). Ape Conservation Physiology: Fecal Glucocorticoid Responses in Wild (*Pongo pygmaeus morio*) following Human Visitation. Plos One.

[44] Seltmann A (2017). Habitat disturbance results in chronic stress and impaired health status in forest-dwelling paleotropical bats. Conservation physiology.

[45] Boonstra R (2013). Reality as the leading cause of stress: rethinking the impact of chronic stress in nature. Funct. Ecol..

[46] Finn HC, Stephens NS (2017). The invisible harm: land clearing is an issue of animal welfare. Wildl. Res..

[47] Hing S, Narayan EJ, Thompson RA, Godfrey SS (2016). The relationship between physiological stress and wildlife disease: consequences for health and conservation. Wildl. Res..

[48] McEwen BS (2005). Stressed or stressed out: what is the difference?. Journal of Psychiatry and Neuroscience.

[49] Demas GE, Adamo SA, French SS (2011). Neuroendocrine-immune crosstalk in vertebrates and invertebrates: implications for host defence. Funct. Ecol..

[50] Chapman CA (2006). Do food availability, parasitism, and stress have synergistic effects on red colobus populations living in forest fragments?. Am. J. Phys. Anthropol..

[51] Brearley G (2013). Wildlife disease prevalence in human‐modified landscapes. Biol. Rev..

[52] Irwin MT, Junge RE, Raharison JL, Samonds KE (2010). Variation in physiological health of diademed sifakas across intact and fragmented forest at Tsinjoarivo, Eastern Madagascar. Am J Primatol.

[53] Johnstone CP, Lill A, Reina RD (2012). Does habitat fragmentation cause stress in the agile antechinus? A haematological approach. Journal of comparative physiology. B, Biochemical, systemic, and environmental physiology.

[54] Saunders DA, Mawson P, Dawson R (2011). The impact of two extreme weather events and other causes of death on Carnaby’s Black Cockatoo: a promise of things to come for a threatened species?. Pac. Conserv. Biol..

[55] Dennison S (2017). Population genetics of the koala (*Phascolarctos cinereus*) in north-eastern New South Wales and south-eastern Queensland. Aust. J. Zool..

[56] Lee KE (2013). Anthropogenic changes to the landscape resulted in colonization of koalas in north‐east New South Wales, Australia. Austral Ecol..

[57] Fahrig L (2003). Effects of habitat fragmentation on biodiversity. Annual review of ecology, evolution, and systematics.

[58] Seabrook LM, McAlpine CA, Phinn SR, Callaghan J, Mitchell D (2003). Landscape legacies: Koala habitat change in Noosa Shire, South-east Queensland. Aust. Zool..

[59] Bender DJ, Fahrig L (2005). Matrix structure obscures the relationship between interpatch movement and patch size and isolation. Ecology.

[60] Martin RO (1985). and decline of a population of the koala, Phascolarctos cinereus, in Victoria. I. Food preference and food tree defoliation. Wildl. Res..

[61] Smith AG (2013). At what spatial scales does resource selection vary? A case study of koalas in a semi-arid region. Austral Ecol..

[62] Mbora DN, McPeek MA (2009). Host density and human activities mediate increased parasite prevalence and richness in primates threatened by habitat loss and fragmentation. J. Anim. Ecol..

[63] Friedman EM, Lawrence DA (2002). Environmental stress mediates changes in neuroimmunological interactions. Toxicol. Sci..

[64] Ibisch PL (2016). A global map of roadless areas and their conservation status. Science.

[65] Govendir M (2012). Plasma concentrations of chloramphenicol after subcutaneous administration to koalas (Phascolarctos cinereus) with chlamydiosis. Journal of veterinary pharmacology and therapeutics.

[66] McCallum H, Dobson A (2002). Disease, habitat fragmentation and conservation. Proceedings of the Royal Society of London B: Biological Sciences.

[67] Short J, Smith A (1994). Mammal decline and recovery in Australia. J. Mammal..

[68] Brooks TM (2002). Habitat loss and extinction in the hotspots of biodiversity. Conserv. Biol..

[69] Ford HA, Barrett GW, Saunders DA, Recher HF (2001). Why have birds in the woodlands of southern Australia declined?. Biol. Conserv..

[70] Lunney D, O’Neill L, Matthews A, Sherwin WB (2002). Modelling mammalian extinction and forecasting recovery: koalas at Iluka (NSW, Australia). Biol. Conserv..

[71] Tobey J, Andrus C, Doyle L, Thompson V, Bercovitch F (2006). Maternal effort and joey growth in koalas (Phascolarctos cinereus). J. Zool..

[72] Bercovitch FB, Tobey JR, Andrus CH, Doyle L (2006). Mating patterns and reproductive success in captive koalas (Phascolarctos cinereus). J. Zool..

[73] Christensen MR (2006). Multiple anthropogenic stressors cause ecological surprises in boreal lakes. Global Change Biol..

[74] Hing S, Narayan EJ, Thompson RCA, Godfrey SS (2017). Identifying factors that influence stress physiology of the woylie, a critically endangered marsupial. J. Zool..

[75] Sih A, Bell AM, Kerby JL (2004). Two stressors are far deadlier than one. Trends Ecol. Evol..

[76] Davies N (2013). Movement patterns of an arboreal marsupial at the edge of its range: a case study of the koala. Movement Ecology.

[77] Narayan, E. J. & Williams, M. Understanding the dynamics of physiological impacts of environmental stressors on Australian marsupials, focus on the koala (Phascolarctos cinereus). BMC Zoology **1**, 10.1186/s40850-016-0004-8 (2016).

